# Supplementary material for: Association of Thymidylate Synthase (TS) Gene Polymorphisms with Incidence and Prognosis of Coronary Artery Disease
Source: Int J Mol Sci. 2023 Aug 9;24(16):12591. doi: 10.3390/ijms241612591 (PMC10454159; doi:10.3390/ijms241612591)
Supplement: Supplementary file 1 [file ijms-24-12591-s001.zip › ijms-2526227-supplementary.pdf]

Table S1. Stratified Analysis of *TS* gene polymorphisms for combined clinical factors in CAD patients and control subjects

		TSER 2R/3R		***TS* 1100T>C**		***TS* 1170A>G**		***TS* 1494ins/del**	
**Variables**	**Model**	**AOR (95% CI)**	***P***	**AOR (95% CI)**	***P***	**AOR (95% CI)**	***P***	**AOR (95% CI)**	***P***
Age									
≤63years	Dominant	0.668 (0.419-1.066)	0.091	1.210 (0.786-1.864)	0.387	0.837 (0.541-1.295)	0.425	0.864 (0.561-1.330)	0.507
	Recessive	1.284 (0.270-6.119)	0.753	1.326 (0.669-2.628)	0.419	0.328 (0.123-0.878)	0.026	0.958 (0.484-1.897)	0.903
>63years	Dominant	1.133 (0.737-1.744)	0.569	1.293 (0.880-1.899)	0.191	0.671 (0.454-0.991)	0.045	0.816 (0.556-1.199)	0.301
	Recessive	1.200 (0.291-4.956)	0.801	1.065 (0.482-2.356)	0.876	0.708 (0.317-1.583)	0.401	0.799 (0.378-1.688)	0.556
Gender									
male	Dominant	0.982 (0.589-1.639)	0.945	1.812 (0.868-3.783)	0.113	0.510 (0.317-0.819)	0.005	0.694 (0.436-1.104)	0.123
	Recessive	1.324 (0.278-6.303)	0.724	1.812 (0.868-3.783)	0.113	0.277 (0.086-0.893)	0.032	1.056 (0.502-2.221)	0.885
female	Dominant	0.907 (0.606-1.358)	0.636	1.440 (0.992-2.089)	0.055	0.890 (0.614-1.290)	0.538	1.043 (0.721-1.509)	0.823
	Recessive	1.349 (0.327-5.556)	0.679	0.805 (0.381-1.698)	0.569	0.613 (0.290-1.295)	0.200	0.873 (0.441-1.726)	0.696
HTN									
No	Dominant	0.668 (0.427-1.045)	0.077	1.324 (0.889-1.972)	0.167	0.915 (0.611-1.371)	0.667	0.794 (0.534-1.181)	0.254
	Recessive	1.895 (0.476-7.540)	0.364	1.226 (0.609-2.470)	0.568	0.558 (0.236-1.322)	0.185	0.833 (0.400-1.732)	0.624
Yes	Dominant	1.310 (0.828-2.072)	0.249	1.307 (0.859-1.989)	0.212	0.555 (0.363-0.848)	0.007	0.966 (0.635-1.469)	0.872
	Recessive	0.523 (0.109-2.503)	0.417	1.283 (0.595-2.768)	0.526	0.445 (0.182-1.088)	0.076	0.991 (0.490-2.006)	0.980
DM									
No	Dominant	0.924 (0.653-1.308)	0.657	1.224 (0.892-1.680)	0.211	0.855 (0.622-1.177)	0.337	0.810 (0.591-1.112)	0.192
	Recessive	0.943 (0.315-2.824)	0.916	1.068 (0.597-1.908)	0.825	0.484 (0.234-1.001)	0.050	0.763 (0.431-1.350)	0.353
Yes	Dominant	0.961 (0.459-2.013)	0.916	1.770 (0.884-3.544)	0.107	0.323 (0.155-0.674)	0.003	1.239 (0.628-2.446)	0.537
	Recessive	N/A	0.998	1.890 (0.556-6.419)	0.308	0.551 (0.162-1.870)	0.339	1.782 (0.531-5.978)	0.350
Lipid									
No	Dominant	0.833 (0.583-1.191)	0.317	1.231 (0.885-1.711)	0.217	0.757 (0.542-1.057)	0.102	0.941 (0.677-1.308)	0.716
	Recessive	0.953 (0.273-3.328)	0.939	1.296 (0.729-2.303)	0.377	0.480 (0.228-1.014)	0.054	1.036 (0.582-1.845)	0.903
Yes	Dominant	1.273 (0.656-2.467)	0.475	1.432 (0.794-2.585)	0.233	0.699 (0.392-1.245)	0.224	0.673 (0.375-1.207)	0.184
	Recessive	1.632 (0.259-10.304)	0.602	0.836 (0.271-2.574)	0.754	0.598 (0.199-1.797)	0.360	0.568 (0.202-1.598)	0.284
Smoking status									
No	Dominant	1.146 (0.788-1.666)	0.477	1.138 (0.809-1.600)	0.457	0.715 (0.507-1.007)	0.055	0.845 (0.601-1.188)	0.333
	Recessive	1.236 (0.358-4.271)	0.738	0.937 (0.483-1.819)	0.848	0.457 (0.219-0.956)	0.038	0.859 (0.461-1.600)	0.632
Yes	Dominant	0.555 (0.301-1.021)	0.058	2.016 (1.159-3.507)	0.013	0.736 (0.421-1.285)	0.281	1.014 (0.592-1.739)	0.959
	Recessive	0.935 (0.139-6.273)	0.945	1.914 (0.847-4.326)	0.119	0.583 (0.183-1.857)	0.361	1.064 (0.457-2.475)	0.886
Homocysteine									
≤11.40μmol/l	Dominant	0.848 (0.590-1.217)	0.370	1.203 (0.864-1.675)	0.274	0.703 (0.502-0.985)	0.041	0.843 (0.606-1.174)	0.312
	Recessive	1.771 (0.578-5.431)	0.317	1.216 (0.675-2.190)	0.515	0.157 (0.052-0.471)	0.001	0.820 (0.464-1.449)	0.494
>11.40μmol/l	Dominant	0.992 (0.508-1.936)	0.981	1.566 (0.852-2.881)	0.149	0.695 (0.383-1.260)	0.231	0.928 (0.509-1.692)	0.808
	Recessive	N/A	0.998	1.370 (0.467-4.023)	0.567	1.063 (0.393-2.874)	0.904	1.528 (0.507-4.607)	0.452
Folate									
≥4.85ng/ml	Dominant	0.787 (0.537-1.155)	0.222	1.350 (0.955-1.909)	0.089	0.679 (0.480-0.961)	0.029	0.949 (0.673-1.340)	0.768
	Recessive	1.539 (0.447-5.294)	0.495	1.236 (0.669-2.286)	0.499	0.425 (0.188-0.961)	0.040	0.978 (0.548-1.746)	0.941
<4.85ng/ml	Dominant	1.274 (0.666-2.436)	0.464	1.248 (0.688-2.265)	0.467	0.913 (0.494-1.686)	0.771	1.015 (0.557-1.848)	0.962
	Recessive	1.609 (0.151-17.161)	0.694	1.430 (0.448-4.566)	0.547	0.345 (0.094-1.265)	0.108	1.229 (0.335-4.504)	0.756

Adjusted by age, gender, hypertension, diabetes mellitus, hyperlipidemia, and smoke.

Table S2. Adjusted odds ratios for CAD risk associated with *TS* gene polymorphisms, combined by clinical factors

**Characteristics**	***TSER***		***TS* 1100T>C**		***TS* 1170A>G**		***TS* 1494 0bp/6bp**	
	**3R3R**	**3R2R+2R2R**	**TT**	**TC+CC**	**AA**	**AG+GG**	**0bo0bp**	**0bp6bp+6bp6bp**
Age								
≤63years	1.000 (reference)	0.668 (0.419-1.066)	1.000 (reference)	1.210 (0.786-1.864)	1.000 (reference)	0.837 (0.541-1.295)	1.000 (reference)	0.864 (0.561-1.330)
>63years	0.876 (0.621-1.234)	1.010 (0.637-1.600)	0.962 (0.628-1.471)	1.290 (0.845-1.967)	1.093 (0.718-1.665)	0.752 (0.493-1.147)	1.033 (0.677-1.575)	0.813 (0.532-1.244)
Gender								
male	1.000 (reference)	0.982 (0.589-1.639)	1.000 (reference)	1.150 (0.727-1.818)	1.000 (reference)	0.510 (0.317-0.819)	1.000 (reference)	0.694 (0.436-1.104)
female	0.768 (0.512-1.155)	0.652 (0.407-1.046)	0.464 (0.275-0.784)	0.623 (0.381-1.018)	0.403 (0.252-0.644)	0.518 (0.321-0.836)	0.441 (0.260-0.748)	0.404 (0.245-0.667)
Hypertension								
No	1.000 (reference)	0.668 (0.427-1.045)	1.000 (reference)	1.324 (0.889-1.972)	1.000 (reference)	0.915 (0.611-1.371)	1.000 (reference)	0.794 (0.534-1.181)
Yes	1.378 (0.979-1.941)	1.661 (1.050-2.630)	1.664 (1.087-2.546)	2.122 (1.401-3.215)	1.797 (1.176-2.745)	1.172 (0.764-1.796)	1.524 (0.992-2.342)	1.406 (0.942-2.099)
Diabetes mellitus								
No	1.000 (reference)	0.924 (0.653-1.308)	1.000 (reference)	1.224 (0.892-1.680)	1.000 (reference)	0.855 (0.622-1.177)	1.000 (reference)	0.810 (0.591-1.112)
Yes	2.865 (1.841-4.458)	2.414 (1.277-4.564)	2.394 (1.405-4.079)	3.996 (2.329-6.855)	4.239 (2.362-7.609)	1.695 (1.013-2.836)	2.421 (1.423-4.117)	2.963 (1.721-5.102)
Hyperlipidemia								
No	1.000 (reference)	0.833 (0.583-1.191)	1.000 (reference)	1.231 (0.885-1.711)	1.000 (reference)	0.757 (0.542-1.057)	1.000 (reference)	0.941 (0.677-1.308)
Yes	1.245 (0.852-1.820)	1.390 (0.770-2.509)	1.372 (0.865-2.175)	1.861 (1.134-3.054)	1.495 (0.932-2.397)	1.037 (0.640-1.680)	1.632 (1.027-2.591)	1.066 (0.657-1.730)
Smoking								
No	1.000 (reference)	1.146 (0.788-1.666)	1.000 (reference)	1.138 (0.809-1.600)	1.000 (reference)	0.715 (0.507-1.007)	1.000 (reference)	0.845 (0.601-1.188)
Yes	0.875 (0.566-1.351)	0.504 (0.278-0.914)	0.407 (0.231-0.717)	0.724 (0.435-1.205)	0.541 (0.336-0.871)	0.484 (0.272-0.860)	0.496 (0.284-0.868)	0.463 (0.277-0.774)
Homocysteine								
≤11.40μmol/l	1.000 (reference)	0.888 (0.622-1.268)	1.000 (reference)	1.180 (0.852-1.635)	1.000 (reference)	0.746 (0.535-1.039)	1.000 (reference)	0.833 (0.602-1.154)
>11.40μmol/l	1.129 (0.752-1.695)	1.106 (0.596-2.050)	0.901 (0.540-1.504)	1.484 (0.905-2.433)	1.038 (0.631-1.707)	0.749 (0.452-1.240)	0.972 (0.584-1.618)	0.948 (0.580-1.550)
Folate								
≥4.85ng/ml	1.000 (reference)	0.791 (0.550-1.137)	1.000 (reference)	1.279 (0.920-1.778)	1.000 (reference)	0.697 (0.501-0.970)	1.000 (reference)	0.831 (0.598-1.153)
<4.85ng/ml	1.590 (1.053-2.399)	2.250 (1.243-4.071)	1.968 (1.182-3.277)	2.621 (1.585-4.333)	1.698 (1.036-2.783)	1.420 (0.859-2.348)	1.621 (0.979-2.685)	1.792 (1.089-2.947)
HDL-cholesterol								
≥40(M), 50(F)	1.000 (reference)	0.468 (0.253-0.865)	1.000 (reference)	0.941 (0.546-1.623)	1.000 (reference)	1.442 (0.819-2.538)	1.000 (reference)	0.648 (0.373-1.125)
<40(M), 50(F)	1.267 (0.789-2.035)	1.585 (0.852-2.950)	1.135 (0.640-2.014)	2.471 (1.294-4.717)	2.556 (1.460-4.477)	1.565 (0.877-2.792)	1.331 (0.735-2.410)	1.387 (0.759-2.535)

Adjusted by age, gender, hypertension, diabetes mellitus, hyperlipidemia, and smoke.
Abbreviations: HDL-cholesterol; high density lipoprotein-cholesterols

Table S3. The 4 site haplotype analysis for *TS* gene polymorphisms in CAD patients and control subjects.

Haplotype	Control (2n=854)	CAD (2n=848)	OR (95% CI)	P^a
TSER/*TS*1100/1170/1494				
3R-T-A-0bp	0.3554	0.3916	1.000 (reference)	
3R-T-A-6bp	0.0250	0.0199	0.778 (0.400-1.514)	0.501
3R-T-G-6bp	0.0020	0.0038	1.373 (0.228-8.279)	1.000
3R-C-A-6bp	0.1744	0.1621	0.836 (0.633-1.106)	0.227
3R-C-G-0bp	-	0.0036	6.411 (0.330-124.700)	0.251
3R-C-G-6bp	-	-	-	-
2R-T-A-0bp	0.0371	0.0242	0.601 (0.339-1.065)	0.087
2R-T-A-6bp	0.0087	0.0073	0.687 (0.236-2.002)	0.593
2R-T-G-0bp	0.0085	0.0188	2.093 (0.849-5.158)	0.136
2R-T-G-6bp	0.0019	0.0015	0.458 (0.041-5.078)	0.609
2R-C-A-0bp	0	-	-	-
2R-C-A-6bp	0.1053	0.0984	0.844 (0.603-1.182)	0.346
2R-C-G-0bp	0.0012	0	0.305 (0.012-7.528)	0.479
2R-C-G-6bp	-	0.0042	8.242 (0.442-153.800)	0.126

Abbreviations: OR, odds ratio; 95% CI, 95% confidence interval; CAD, coronary artery disease; TSER, thymidylate synthase enhancer region; TS, thymidylate synthase; FDR, false discovery rate.

[a] *P*-value calculated by chi-square test and fisher's exact test. The *P*-value<0.05 showed the bold type in Table 3.

Table S4. The 3 site haplotype analysis for *TS* gene polymorphisms CAD patients and control subjects.

**Haplotype**	**Control (2n=854)**	**CAD (2n=848)**	**OR (95% CI)**	***P*[a]**
TSER/*TS*1100/1170				
3R-T-A	0.3781	0.4148	1.000 (Reference)	
3R-C-A	0.1798	0.2118	1.073 (0.825 - 1.395)	0.601
3R-C-G	-	0.0058	10.100 (0.556 - 183.400)	0.063
2R-T-G	0.01	0.0218	1.835 (0.813 - 4.143)	0.117
2R-C-A	0.1026	0.0975	0.865 (0.619 - 1.211)	0.399
2R-C-G	0.0022	0.0045	1.835 (0.334 - 10.090)	0.688
TSER/*TS*1100/1494				
3R-T-0bp	0.6336	0.6049	1.000 (Reference)	
3R-T-6bp	0.0253	0.0238	0.959 (0.517 - 1.778)	0.893
3R-C-6bp	0.1746	0.1618	0.970 (0.746 - 1.260)	0.817
2R-T-0bp	0.0454	0.0429	0.973 (0.609 - 1.556)	0.910
2R-T-6bp	0.0111	0.0088	0.820 (0.303 - 2.219)	0.694
2R-C-0bp	0	0	-	-
2R-C-6bp	0.1063	0.1028	1.008 (0.734 - 1.385)	0.960
TSER/*TS*1170/1494				
3R-A-0bp	0.3592	0.4487	1.000 (Reference)	
3R-G-6bp	0.0034	0.0067	1.616 (0.401 - 6.516)	0.738
2R-G-0bp	0.0098	0.0202	1.717 (0.731 - 4.032)	0.183
*TS*1100/1170/1494				
T-A-0bp	0.392	0.4153	1.000 (Reference)	
T-A-6bp	0.0331	0.0273	0.782 (0.441 - 1.384)	0.395
T-G-6bp	0.0033	0.0053	1.269 (0.282 - 5.714)	1.000
C-A-6bp	0.2809	0.2612	0.876 (0.692 - 1.110)	0.273
C-G-0bp	0.0012	0.004	2.855 (0.295 - 27.600)	0.625
C-G-6bp	-	0.0034	6.662 (0.343 - 129.600)	0.250

Abbreviations: OR, odds ratio; 95% CI, 95% confidence interval; CAD, coronary artery disease; TSER, thymidylate synthase enhancer region; TS, thymidylate synthase; FDR, false discovery rate.

[a] *P*-value calculated by chi-square test and fisher's exact test. The *P*-value<0.05 showed the bold type in Table 3.

Table S5. The 2 site haplotype analysis for *TS* gene polymorphisms in CAD patients and control subjects.

Haplotype	Control (2n=854)	CAD (2n=848)	OR (95% CI)	P^a
TSER/*TS*1100				
3R-T	0.6569	0.6282	1.000 (Reference)	
3R-C	0.1804	0.2173	1.258 (0.985 - 1.606)	0.065
2R-T	0.0586	0.0522	0.926 (0.607 - 1.413)	0.722
2R-C	0.1042	0.1023	1.029 (0.748 - 1.415)	0.861
TSER/*TS* 1494				
3R-0bp	0.6371	0.66	1.000 (Reference)	
3R-6bp	0.2002	0.1855	0.892 (0.697 - 1.141)	0.363
2R-0bp	0.0456	0.0428	0.897 (0.561 - 1.432)	0.648
2R-6bp	0.1172	0.1117	0.923 (0.681 - 1.251)	0.605
TS 1100/1170				
T-A	0.4259	0.4452	1.000 (Reference)	
C-A	0.2826	0.3095	1.047 (0.835 - 1.313)	0.692
TS 1100/1494				
T-0bp	0.679	0.6478	1.000 (Reference)	
T-6bp	0.0364	0.0326	0.954 (0.565 - 1.612)	0.861
C-6bp	0.2809	0.2645	0.986 (0.794 - 1.224)	0.899

Abbreviations: OR, odds ratio; 95% CI, 95% confidence interval; CAD, coronary artery disease; TSER, thymidylate synthase enhancer region; TS, thymidylate synthase; FDR, false discovery rate.

[a] *P*-value calculated by chi-square test and fisher's exact test. The *P*-value<0.05 showed the bold type in Table 3.

Table S6. Genotype combination analyses for the *TS* gene polymorphisms in CAD patients and controls

Genotype combinations	Controls (n=427)	CAD (n=424)	AOR (95% CI)	P^*
TSER/*TS* 1100T>C				
3R3R/TT	182 (42.6)	166 (39.2)	1.000 (reference)	
3R3R/TC	99 (23.2)	110 (25.9)	1.259 (0.880 - 1.801)	0.207
3R3R/CC	15 (3.5)	25 (5.9)	1.927 (0.951 - 3.906)	0.069
2R3R/TT	33 (7.7)	27 (6.4)	0.847 (0.467 - 1.534)	0.583
2R3R/TC	75 (17.6)	75 (17.7)	1.142 (0.753 - 1.732)	0.533
2R3R/CC	15 (3.5)	13 (3.1)	0.705 (0.301 - 1.653)	0.421
2R2R/TT	2 (0.5)	1 (0.2)	0.652 (0.053 - 8.053)	0.739
2R2R/TC	3 (0.7)	4 (0.9)	1.718 (0.365 - 8.079)	0.493
2R2R/CC	3 (0.7)	3 (0.7)	1.021 (0.174 - 6.003)	0.982
TSER/*TS* 1170A>G				
3R3R/AA	130 (30.4)	164 (38.7)	1.000 (reference)	
2R3R/AG	46 (10.8)	47 (11.1)	0.731 (0.435 - 1.229)	0.238
2R3R/GG	3 (0.7)	3 (0.7)	0.865 (0.161 - 4.651)	0.866
2R2R/AA	7 (1.6)	5 (1.2)	0.611 (0.169 - 2.214)	0.453
2R2R/AG	1 (0.2)	3 (0.7)	2.368 (0.235 - 23.905)	0.465
2R2R/GG	0 (0.0)	0 (0.0)	-	-
TSER/*TS* 1494ins>del				
3R3R/0bp0bp	170 (39.8)	187 (44.1)	1.000 (reference)	
3R3R/0bp6bp	107 (25.1)	95 (22.4)	0.819 (0.572 - 1.173)	0.277
3R3R/6bp6bp	19 (4.4)	19 (4.5)	0.971 (0.485 - 1.945)	0.934
2R3R/0bp0bp	24 (5.6)	23 (5.4)	0.802 (0.419 - 1.539)	0.508
2R3R/0bp6bp	82 (19.2)	75 (17.7)	0.881 (0.587 - 1.323)	0.542
2R3R/6bp6bp	17 (4.0)	17 (4.0)	0.709 (0.328 - 1.534)	0.382
2R2R/0bp0bp	1 (0.2)	1 (0.2)	1.581 (0.083 - 30.228)	0.761
2R2R/0bp6bp	4 (0.9)	4 (0.9)	0.924 (0.221 - 3.873)	0.914
2R2R/6bp6bp	3 (0.7)	3 (0.7)	0.925 (0.157 - 5.460)	0.932
TS 1100T>C/*TS* 1170A>G				
TT/AA	70 (16.4)	86 (20.3)	1.000 (reference)	
TC/AA	109 (25.5)	109 (25.7)	0.866 (0.550 - 1.363)	0.534
TC/AG	68 (15.9)	78 (18.4)	0.837 (0.511 - 1.370)	0.479
TC/GG	0 (0.0)	2 (0.5)	-	0.995
CC/AA	32 (7.5)	39 (9.2)	0.841 (0.442 - 1.602)	0.599
CC/AG	1 (0.2)	2 (0.5)	1.973 (0.141 - 27.593)	0.614
CC/GG	0 (0.0)	0 (0.0)	-	-
TS 1100T>C/*TS* 1494ins>del				
TT/0bp0bp	194 (45.4)	180 (42.5)	1.000 (reference)	
TT/0bp6bp	18 (4.2)	8 (1.9)	0.492 (0.199 - 1.214)	0.124
TT/6bp6bp	5 (1.2)	6 (1.4)	1.513 (0.414 - 5.528)	0.531
TC/0bp6bp	173 (40.5)	162 (38.2)	1.053 (0.772 - 1.436)	0.745
TC/6bp6bp	3 (0.7)	6 (1.4)	1.853 (0.432 - 7.953)	0.407
CC/0bp0bp	0 (0.0)	10 (2.4)	-	0.995
CC/0bp6bp	2 (0.5)	4 (0.9)	2.752 (0.461 - 16.411)	0.267
CC/6bp6bp	31 (7.3)	27 (6.4)	0.871 (0.479 - 1.582)	0.650
TS 1170A>G/*TS* 1494ins>del				
AA/0bp0bp	57 (13.3)	101 (23.8)	1.000 (reference)	
AG/6bp6bp	2 (0.5)	3 (0.7)	1.140 (0.145 - 8.972)	0.901
GG/0bp6bp	0 (0.0)	2 (0.5)	-	0.995
GG/6bp6bp	0 (0.0)	0 (0.0)	-	-

Abbreviation: AOR, adjusted odds ratio; 95% CI, 95% confidence interval; CAD, coronary artery disease; TS, thymidylate synthase. *Adjusted by age, sex, hypertension, diabetes mellitus, hyperlipidemia, and smoking. The *P*-values <0.05 showed the bold type in Table 4

Table S7. Baseline characteristics between controls and CAD patients in sample 1 and 2

Characteristic	Sample 1*			Sample 2**		
	Controls (n=288)	CAD patients (n=166)	*P*	Controls (n=139)	CAD patients (n=251)	*P*
Male (n, %)	95 (33.0)	58 (34.9)	0.766	73 (52.5)	96 (38.2)	0.091
Age (years, mean ± SD)	61.22±11.59	64.13±9.91	0.019[a]	61.88±11.42	61.47±10.42	0.724
Hypertension (n, %)	114 (39.6)	92 (55.4)	0.048	57 (41.0)	133 (53.0)	0.178
Diabetes mellitus (n, %)	36 (12.5)	62 (37.3)	<0.0001	15 (10.8)	55 (21.9)	0.020
Hyperlipidemia (n, %)	68 (23.6)	44 (26.5)	0.593	29 (20.9)	71 (28.3)	0.212
Smoking (n, %)	75 (26.0)	45 (27.1)	0.850	61 (43.9)	61 (24.3)	0.005
Body mass index (kg/cm2, mean ± SD)	24.27±3.34	24.85±3.15	0.096	24.26±3.35	25.44±3.13	0.001
Fasting blood sugar (mg/dL, mean ± SD)	110.42±35.02	153.61±70.51	<0.0001[a]	115.11±31.55	133.80±58.45	<0.001[a]
HbA1c (%, mean ± SD)	6.58±1.61	7.09±1.80	0.143	5.87±0.82	6.49±3.43	0.101[a]
HDL-C (mg/dl, mean ± SD)	46.18±16.18	41.69±10.17	0.056[a]	47.78±11.47	45.11±12.27	0.082
LDL-C (mg/dl, mean ± SD)	128.73±52.24	116.35±37.46	0.161[a]	114.70±32.35	109.28±38.39	0.249
Total cholesterol (mg/dl, mean ± SD)	196.25±40.05	189.03±45.68	0.023[a]	189.59±33.74	184.64±44.59	0.124[a]
Triglyceride (mg/dl, mean ± SD)	149.07±91.82	164.88±99.82	0.092	128.38±73.96	150.36±95.68	0.006[a]
Hcy (μmol/L, mean ± SD)	9.41±3.37	10.62±4.21	0.001[a]	10.50±4.74	9.52±5.57	0.001[a]
Folate (nmol/L, mean ± SD)	9.28±8.31	10.51±14.34	0.551[a]	8.87±7.17	7.59±5.27	0.019[a]

P-values were calculated by two-sided t-test for continuous variables and chi-square test for categorical variables. [a] P-values were calculated by Mann-Whitney test for continuous variables. CAD, coronary artery disease; SD, standard deviation; HDL-C, high density lipoprotein-cholesterol; LDL-C, low density lipoprotein-cholesterol; Hcy, homocysteine; HbA1c, hemoglobin A1c. * Sample 1 was recruited from 2000 to 2006. ** Sample 2 was recruited from 2007 to 2012.

Table S8. Genotype frequencies of *TS* gene polymorphisms between CAD patients and control subjects in samples 1 and 2

Genotypes	Sample 1**				Sample 2***			
	**Controls (n=288)**	**CAD patients (n=166)**	**AOR (95% CI)***	***P*[a]**	**Controls (n=139)**	**CAD patients (n=251)**	**AOR (95% CI)***	***P*[a]**
TSER 2R/3R								
3R3R	205 (71.2)	119 (71.7)	1.000 (reference)		91 (65.5)	175 (69.7)	1.000 (reference)	
2R3R	80 (27.8)	46 (27.7)	1.044 (0.654 - 1.665)	0.857	43 (30.9)	69 (27.5)	0.799 (0.489 - 1.305)	0.370
2R2R	3 (1.0)	1 (0.6)	1.129 (0.100 - 12.707)	0.922	5 (3.6)	7 (2.8)	0.786 (0.233 - 2.658)	0.699
Dominant model			1.045 (0.658 - 1.660)	0.852			0.793 (0.495 - 1.271)	0.336
Recessive model			1.042 (0.095 - 11.393)	0.973			0.794 (0.235 - 2.688)	0.711
TS 1100T>C								
TT	150 (52.1)	63 (38.0)	1.000 (reference)		67 (48.2)	126 (50.2)	1.000 (reference)	
TC	118 (41.0)	80 (48.2)	**1.800 (1.156 - 2.804)**	**0.009**	59 (42.4)	107 (42.6)	0.964 (0.608 - 1.529)	0.876
CC	20 (6.9)	23 (13.9)	**2.741 (1.300 - 5.783)**	**0.008**	13 (9.4)	18 (7.2)	0.732 (0.302 - 1.774)	0.490
Dominant model			**1.949 (1.274 - 2.983)**	**0.002**			0.937 (0.602 - 1.458)	0.773
Recessive model			**2.044 (1.029 - 4.061)**	**0.041**			0.790 (0.350 - 1.783)	0.570
TS 1170A>G								
AA	140 (48.6)	112 (67.5)	1.000 (reference)		71 (51.1)	118 (47.0)	1.000 (reference)	
AG	124 (43.1)	47 (28.3)	**0.470 (0.298 - 0.744)**	**0.001**	59 (42.4)	123 (49.0)	1.022 (0.642 - 1.626)	0.928
GG	24 (8.3)	7 (4.2)	**0.341 (0.129 - 0.900)**	**0.030**	9 (6.5)	10 (4.0)	0.492 (0.177 - 1.365)	0.173
Dominant model			**0.447 (0.290 - 0.691)**	**0.0003**			0.965 (0.617 - 1.511)	0.877
Recessive model			0.481 (0.191 - 1.210)	0.120			0.575 (0.217 - 1.525)	0.266
TS 1494ins/del								
0bp0bp	135 (46.9)	78 (47.0)	1.000 (reference)		60 (43.2)	128 (51.0)	1.000 (reference)	
0bp6bp	127 (44.1)	68 (41.0)	0.978 (0.632 - 1.512)	0.920	66 (47.5)	104 (41.4)	0.750 (0.473 - 1.191)	0.223
6bp6bp	26 (9.0)	20 (12.0)	1.286 (0.633 - 2.611)	0.487	13 (9.4)	19 (7.6)	0.685 (0.286 - 1.642)	0.397
Dominant model			1.031 (0.681 - 1.560)	0.887			0.751 (0.482 - 1.170)	0.205
Recessive model			1.270 (0.651 - 2.477)	0.484			0.839 (0.376 - 1.874)	0.669

AOR, adjusted odds ratio; 95% CI, 95% confidence interval; CAD, coronary artery disease; TSER, thymidylate synthase enhancer region; TS, thymidylate synthase; HWE, Hardy–Weinberg equilibrium. The *P*-value<0.05 showed the bold type in supplementary table 6.

*The AOR on the basis of risk factors such as age, gender, hypertension, diabetes mellitus, hyperlipidemia, and smoking.

[a] *P*-value calculated by multivariable logistic regression.

** Sample 1 was recruited from 2000 to 2006. *** Sample 2 was recruited from 2007 to 2012.

Table S9. Genotype frequencies of *TS* gene polymorphisms between stable CAD, ACS patients and control subjects

Genotypes	Controls (n=427)	Stable CAD (n=205)	AOR (95% CI)*	*P*[a]	ACS (n=219)	AOR (95% CI)*	*P*[a]
TSER 2R/3R							
3R3R	296 (69.3)	148 (72.2)	1.000 (reference)		153 (69.9)	1.000 (reference)	
2R3R	123 (28.8)	52 (25.4)	0.804 (0.536 - 1.205)	0.291	63 (28.8)	1.054 (0.713 - 1.560)	0.791
2R2R	8 (1.9)	5 (2.4)	1.480 (0.463 - 4.732)	0.509	3 (1.4)	0.701 (0.167 - 2.941)	0.627
Dominant model			0.846 (0.573 - 1.250)	0.402		1.033 (0.703 - 1.517)	0.869
Recessive model			1.544 (0.485 - 4.921)	0.463		0.699 (0.167 - 2.930)	0.624
TS 1100T>C							
TT	217 (50.8)	93 (45.4)	1.000 (reference)		101 (46.1)	1.000 (reference)	
TC	177 (41.5)	96 (46.8)	1.259 (0.873 - 1.814)	0.217	93 (42.5)	1.211 (0.836 - 1.754)	0.311
CC	33 (7.7)	16 (7.8)	0.984 (0.486 - 1.992)	0.965	25 (11.4)	1.654 (0.892 - 3.069)	0.110
Dominant model			1.220 (0.858 - 1.735)	0.267		1.290 (0.907 - 1.835)	0.156
Recessive model			0.911 (0.466 - 1.782)	0.786		1.554 (0.867 - 2.784)	0.139
TS 1170A>G							
AA	211 (49.4)	104 (50.7)	1.000 (reference)		130 (59.4)	1.000 (reference)	
AG	183 (42.9)	93 (45.4)	0.936 (0.650 - 1.348)	0.721	79 (36.1)	**0.605 (0.416 - 0.881)**	**0.009**
GG	33 (7.7)	8 (3.9)	0.459 (0.197 - 1.069)	0.071	10 (4.6)	**0.428 (0.191 - 0.957)**	**0.039**
Dominant model			0.863 (0.606 - 1.228)	0.412		**0.579 (0.404 - 0.830)**	**0.003**
Recessive model			0.483 (0.214 - 1.088)	0.079		0.553 (0.258 - 1.187)	0.129
TS 1494ins/del							
0bp0bp	195 (45.7)	94 (45.9)	1.000 (reference)		117 (53.4)	1.000 (reference)	
0bp6bp	193 (45.2)	94 (45.9)	1.047 (0.726 - 1.509)	0.807	80 (36.5)	0.707 (0.488 - 1.026)	0.068
6bp6bp	39 (9.1)	17 (8.3)	0.775 (0.392 - 1.531)	0.463	22 (10.0)	0.968 (0.524 - 1.787)	0.916
Dominant model			0.998 (0.702 - 1.418)	0.991		0.752 (0.530 - 1.069)	0.112
Recessive model			0.784 (0.412 - 1.490)	0.457		1.132 (0.632 - 2.027)	0.677

AOR, adjusted odds ratio; 95% CI, 95% confidence interval; CAD, coronary artery disease; ACS, acute coronary syndrome; TSER, thymidylate synthase enhancer region; TS, thymidylate synthase; HWE, Hardy–Weinberg equilibrium.

*The AOR on the basis of risk factors such as age, gender, hypertension, diabetes mellitus, hyperlipidemia, and smoking.

[a] *P*-value calculated by multivariable logistic regression.

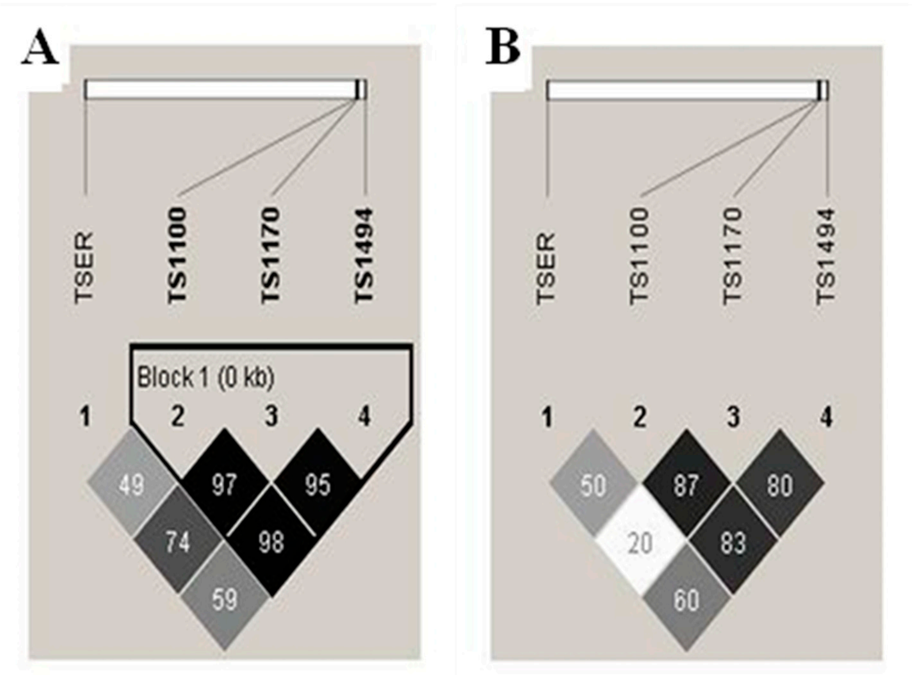

Figure S1. LD patterns of *TS* gene polymorphisms. The values in the squares denote LD between single markers. (A) Control subjects exhibited strong LD block that *TS* 1100/1170/1494 haplotype. (B) Patients with CAD not exhibited LD block. Dark squares indicate high r2 values and light squares indicate low r2 values. LD, linkage disequilibrium; *TS*, thymidylate synthase; CAD, coronary artery disease.
